# Exercise Improves the Coordination of the Mitochondrial Unfolded Protein Response and Mitophagy in Aging Skeletal Muscle

**DOI:** 10.3390/life13041006

**Published:** 2023-04-13

**Authors:** Yan Wang, Jialin Li, Ziyi Zhang, Runzi Wang, Hai Bo, Yong Zhang

**Affiliations:** 1 Tianjin Key Laboratory of Exercise Physiology and Sports Medicine, School of Exercise and Health, Tianjin University of Sport, Tianjin 301617, China; 2 School of Physical Education, Guangdong Institute of Petrochemical Technology, Maoming 525000, China; 3 Department of Military Training Medicines, Logistics University of Chinese People’s Armed Police Force, Tianjin 300162, China

**Keywords:** UPRmt, mitophagy, mitochondrial homeostasis, mitochondrial network, reactive oxygen species, endurance exercise, aging, skeletal muscle

## Abstract

The mitochondrial unfolded protein response (UPRmt) and mitophagy are two mitochondrial quality control (MQC) systems that work at the molecular and organelle levels, respectively, to maintain mitochondrial homeostasis. Under stress conditions, these two processes are simultaneously activated and compensate for each other when one process is insufficient, indicating mechanistic coordination between the UPRmt and mitophagy that is likely controlled by common upstream signals. This review focuses on the molecular signals regulating this coordination and presents evidence showing that this coordination mechanism is impaired during aging and promoted by exercise. Furthermore, the bidirectional regulation of reactive oxygen species (ROS) and AMPK in modulating this mechanism is discussed. The hierarchical surveillance network of MQC can be targeted by exercise-derived ROS to attenuate aging, which offers a molecular basis for potential therapeutic interventions for sarcopenia.

## 1. Introduction

Skeletal muscles are multifunctional organs that exhibit high plasticity and adaptability in response to changes in exercise, nutrition, and stress. The age-related decline in muscle mass, strength, and function, known as sarcopenia, is well-established and has been linked to a variety of negative health outcomes, including loss of mobility, metabolic dysfunction, chronic disease susceptibility, and increased mortality risk [1,2]. The underlying mechanisms driving sarcopenia are not fully understood, but it is widely recognized that disruption of mitochondrial homeostasis plays a major role [3]. Mitochondrial quality control (MQC) is critical for maintaining mitochondrial homeostasis, which encompasses multiple mechanisms at the molecular and organelle levels, such as antioxidants, DNA repair systems, protein import, the mitochondrial unfolded protein response (UPRmt), mitochondrial fusion and fission, mitophagy, and mitochondrial biogenesis [4]. Reductions in MQC are associated with dysregulation of mitochondrial homeostasis and impaired mitochondrial function, leading to aging and sarcopenia [5]. Disorganized mitochondria can further exacerbate these negative effects by promoting cell senescence and death via energy depletion, disruption of calcium metabolism, ferroptosis, apoptosis, and other mechanisms.

Regular physical exercise is an integral component of human life that exerts significant effects on preserving health and preventing muscle-related disorders. The soundness and operational efficiency of skeletal muscle mitochondria are crucial for sustaining physical activity because they are responsible for providing energy through oxidative phosphorylation in skeletal muscle cells. Apart from their well-established role as energy-producing organelles, mitochondria also play a vital role in regulating calcium homeostasis, a critical process for muscle contraction. Additionally, mitochondria are key signal-regulating organelles that produce reactive oxygen species (ROS) that modulate redox signaling and regulate energy-sensitive pathways such as AMPK during exercise. Mitochondria exhibit a high degree of plasticity during exercise by adapting their volume, structure, and function, such as augmenting mitochondrial biogenesis, activating mitophagy, and boosting the capacity for protein import into mitochondria and UPRmt [6,7]. Therefore, regular exercise presents a plausible approach that has the potential to ameliorate MQC and attenuate the decline in skeletal muscle that typically occurs with aging.

The UPRmt and mitophagy are integrated into a hierarchical surveillance network of MQC by counteracting the adverse effects caused by mitochondrial damage [8]. The UPRmt is proposed to be involved in mediating exercise-induced adaptations in muscles and may even precede changes in mitochondrial import, autophagy, and biogenesis [6,9]. Under normal conditions, the UPRmt responds to the accumulation of misfolded or damaged proteins to maintain mitochondrial proteostasis by promoting the transcription of intraorganellar chaperones, proteases, and antioxidants. This mechanism is referred to as molecular quality control [10]. However, the UPRmt has a limited capacity to eliminate defective mitochondrial components and becomes overwhelmed if the damage continues or increases. In such cases, mitophagy acts as a second line of defense and eliminates damaged mitochondria [11]. This mechanism is referred to as organelle quality control.

In recent years, various signaling pathways have been found to regulate the mitophagy-mediated degradation of mitochondria. Interestingly, these pathways also activate the UPRmt, suggesting a possible coordinated control of these two systems that are responsible for the maintenance of mitochondrial quality. The available evidence demonstrates that under stress conditions, the UPRmt and mitophagy are simultaneously activated and compensate for each other when one system becomes insufficient. The coordination of the UPRmt and mitophagy is probably controlled by common upstream signaling pathways, which are part of a complex network of molecular and organelle systems involved in MQC mechanisms. Because dysfunction of the UPRmt and mitophagy is associated with certain diseases, including sarcopenia, identifying the molecular targets involved in this coordination mechanism may hold therapeutic potential for muscle-related disorders [12].

Based on recent research evidence, the exercise-induced activation of UPRmt and mitophagy has been shown to improve muscle quality in elderly individuals. For instance, exercise has been demonstrated to enhance the muscle mitochondrial quality by improving UPRmt activation, which is associated with improvements in exercise performance, including increased muscle strength, maximum running speed, and running distance in older adults [13]. Additionally, the overexpression of Parkin-mediated mitophagy has been shown to protect against oxidative stress, increase mitochondrial contents, and promote enzyme activity and muscle mass in both young and old individuals [14]. Because the UPRmt and mitophagy play a crucial role in regulating the integrity of aging mitochondria, understanding how moderate-intensity muscle contractile activity stimulates and restores these systems to prevent aging is essential.

This review provides a comprehensive analysis of the multifaceted biological functions of the UPRmt and mitophagy and places a particular emphasis on their coordination mechanism and how it is impacted by aging and exercise. This paper also explores the bidirectional regulation of ROS induced by exercise or aging and its impact on several signaling pathways that are involved in this coordinated mechanism. Understanding the intricate mechanisms underlying mitochondrial homeostasis may provide valuable insights into the development of therapeutic strategies for the treatment of age-related muscle wasting and for promoting muscle health throughout the human lifespan.

## 2. MQC

MQC is the collective term for mechanisms that maintain the quantity, morphology, and function of mitochondria, which are primarily mediated by the interplay among protein import, UPRmt, mitochondrial fusion and fission, mitophagy, and mitochondrial biogenesis [8,15]. These systems are highly interconnected via intricate molecular machinery and are now recognized as master regulators of mitochondrial homeostasis [16].

### 2.1. UPRmt

**Figure 1:**
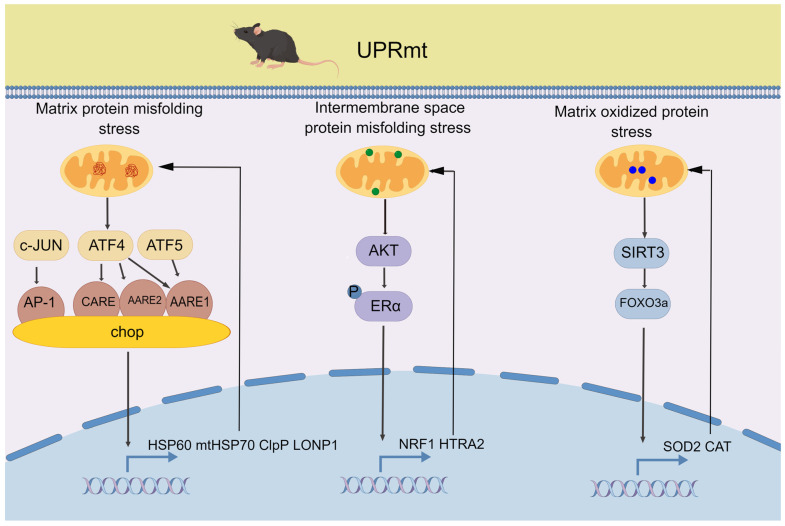
The three main pathways of mammalian UPRmt. In the mitochondrial unfolded protein pathway, mitochondrial stress leads to the accumulation of unfolded or misfolded proteins in the matrix, which triggers the activation of transcription factors including c-Jun, ATF4, ATF5, and chop. These factors induce the expression of genes involved in protein folding and degradation, leading to the restoration of proteostasis. In the mitochondrial intermembrane space pathway, in response to OMM or inner membrane (IMM) damage, the transcription factors ERα and NRF1 are activated to promote the expression of genes involved in mitochondrial membrane repair and biogenesis. Additionally, the protease HTRA2 is activated to degrade damaged mitochondrial proteins. In the mitochondrial matrix oxidized protein pathway, the transcription factor SIRT3 is activated in response to oxidative stress and regulates the activity of FOXO3a, which in turn regulates the expression of genes involved in mitochondrial antioxidant defense and protein quality control, such as SOD2 and CAT, the antioxidant enzymes that detoxify reactive oxygen species in the mitochondrial matrix.

Mitochondrial proteome aberrations, which are induced by stressful stimuli, generate biological signals that are transmitted to the nucleus, where they rapidly trigger extensive transcriptional responses and thereby prevent the accumulation of damaged proteins. This feedback loop is referred to as the UPRmt and plays a vital role in the maintenance of mitochondrial homeostasis and cellular health [17]. In mammals, at least three resident axes are involved in the UPRmt (Figure 1). First, transcription in response to mitochondrial matrix stress requires the C/EBP transcription factor CHOP, which is presumably induced by c-Jun, ATF4, and ATF5. c-Jun activates CHOP through an AP-1 site [18], whereas ATF4 invokes CHOP promoter activity via cis-acting AARE1 and AARE2 as well as the composite CARE [19]. ATF5 mediates the UPRmt in a manner similar to ATFS-1 in worms and activates the CHOP gene promoter through the AARE1 site [20,21]. CHOP triggers the classical UPRmt pathway, which involves the transcription of genes encoding mitochondrial chaperones such as HSP60/HSP10 and mtHSP70 that assist in protein folding and assembly and mitochondrial proteases such as CLpP and LONP1 that degrade excess or damaged proteins [22]. Second, the UPRmt can be triggered by protein misfolding in the mitochondrial intermembrane space, leading to ROS overproduction and subsequent activation of Akt and phosphorylation of ERα. Once phosphorylated, ERα upregulates the expression of NRF1, which enhances proteasome activity, and mitochondrial serine protease HTRA2 (aka Omi), which cleaves aggregated proteins [23]. Third, FOXO3a is deacylated in a SIRT3-dependent manner in response to oxidative protein folding in the mitochondrial matrix, resulting in its accumulation in the nucleus. Activated FOXO3a promotes the antioxidant machinery proteins SOD2 and catalase, which can repair moderately damaged mitochondria [24]. The integrated stress response (ISR) plays a critical role in metabolic integration and extensive crosstalk among signaling pathways in mammals. This response is induced through phosphorylated eIF2α, leading to preferential transcription of genes with upstream open reading frames, such as ATF4, ATF5, and CHOP, which activate the UPRmt pathway [21]. Recent studies found that HSF1 binds to UPRmt chaperone promoters and induces their transcription through direct or indirect phosphorylation of Ser326 in response to impaired mitochondrial proteostasis, which highlights that HSF1 is a novel transcription factor needed for UPRmt activation during mitochondrial stress [17]. Furthermore, UPRmt activation can be triggered by other stresses, such as oxidative phosphorylation impairment and metabolite disturbances, via the aforementioned pathways [25]. Interestingly, the UPRmt protease can import unfolded proteins from the cytoplasm and degrade them, a process known as “mitochondria as guardians in the cytoplasm (MAGIC)” [26]. This discovery suggests that the UPRmt not only protects organelle homeostasis but also cellular proteostasis, although the underlying mechanisms remain unclear.

### 2.2. Mitophagy

**Figure 2:**
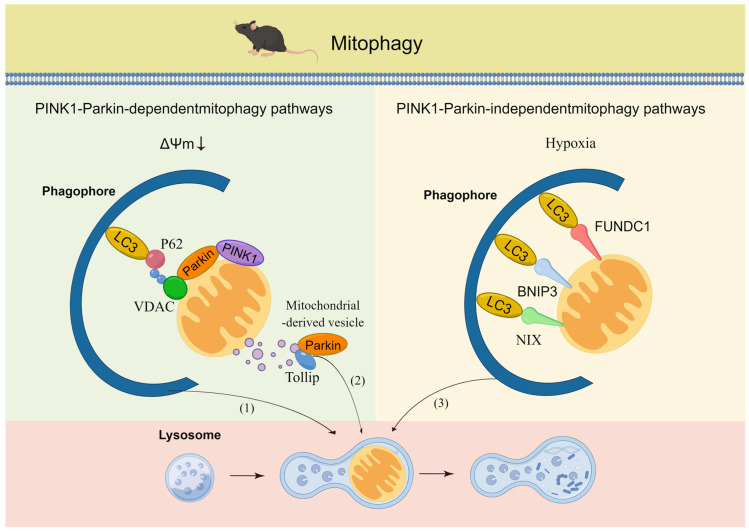
PINK1-Parkin-dependent and independent pathways of mitophagy. PINK1-Parkin-dependent pathway: (1) PINK1-Parkin pathway. When the mitochondrial membrane potential is decreased, PINK1 accumulates on the outer mitochondrial membrane and recruits Parkin. Parkin then ubiquitinates various mitochondrial proteins, such as VDAC, which leads to the recognition and engulfment of mitochondria by autophagosomes. (2) Mitochondrial-derived vesicle (MDV) pathway: this pathway involves the formation of vesicles that bud off from the outer mitochondrial membrane and enclose a portion of the mitochondrial matrix. These vesicles are partly dependent on Parkin and then recognized and engulfed by autophagosomes. This pathway is important for the removal of small portions of mitochondria. PINK1-Parkin-independent pathway: (3) This pathway involves the recruitment of receptor proteins, such as BNIP3, NIX, and FUNDC1, to damaged mitochondria. These proteins bind to LC3 on the autophagosome, leading to the engulfment of mitochondria. This pathway is important for the removal of damaged mitochondria under hypoxia or when the PINK1-Parkin pathway is not functional.

Mitophagy is a selective autophagy mechanism that eliminates depolarized or superfluous mitochondria via the autophagosome–lysosome pathway in response to different cellular energy states, stresses, and signaling cues [27]. The classical and prevalent mitophagy pathways in mammals rely on PTEN-induced putative protein kinase 1 (PINK1) and the E3-ubiquitin ligase Parkin [28]. Upon mitochondrial membrane depolarization, PINK1 accumulates on the outer mitochondrial membrane (OMM), where it primarily recruits and activates Parkin. Parkin ubiquitinates specific fusion proteins located in the OMM and thus promotes the segregation of dysfunctional from functional mitochondria and flags them for removal. Furthermore, autophagosome formation is facilitated by activated Unc-51-like kinase 1 (ULK1) along with microtubule-associated protein light chain 3A (LC3). Additionally, PINK1-mediated mitophagy independent of Parkin has been observed, particularly in a pancreatic cancer cell model under glycolytic suppression [29]. In contrast, the mitophagy response to changes in the hypoxia levels and energy demand is reliant on mitochondrial membrane receptors, even in the absence of mitochondrial membrane depolarization [30]. This regulatory process involves mitochondrial receptor proteins located in the OMM, such as BNIP3, BNIP3L/NIX, and FUNDC1, which carry an LIR motif that directly binds to LC3 or GABARAP on autophagosomes [28]. In addition to the aforementioned mitophagy pathways, other mechanisms have also been identified, and these include the mitochondrial-derived vesicle (MDV) pathway, which involves the budding of small vesicles from the OMM that are then engulfed by the lysosome. The MDV pathway is dependent on Parkin and can be activated by mitochondrial stress, such as hypoxia or mitochondrial DNA damage [31]. These critical mitophagy pathways are activated by distinct intracellular and/or extracellular signals that overlap with one another to efficiently maintain mitochondrial homeostasis (Figure 2). To accurately quantify mitophagy across different tissues, a multitude of transgenic mouse models have been created, including mito-QC, mito-timer, and mito-keima, which allow for the visualization and measurement of various mitochondrial properties in vivo. The mito-QC transgenic mouse model utilizes a fluorescent protein, mCherry fused with a green fluorescent protein (GFP), which is specifically targeted to the mitochondria, providing a means to observe mitochondrial quality control mechanisms [32]. The mito-timer transgenic mouse model, on the other hand, expresses a mitochondria-targeted fluorescent protein that undergoes a color change over time, with the rate of color change being indicative of the age of the mitochondrion, thereby enabling the measurement of mitochondrial turnover and aging in vivo [33]. Similarly, the mito-keima transgenic mouse model employs a pH-sensitive fluorescent protein that is also directed to the mitochondria, allowing for the measurement of mitochondrial turnover through the monitoring of the green signal’s transition to red as mitochondria are engulfed by autophagosomes and delivered to lysosomes for degradation [34]. In summary, these transgenic mouse models represent invaluable tools for investigating mitophagy in vivo.

## 3. Coordination Mechanism of the UPRmt and Mitophagy

### 3.1. Relationship between the UPRmt and Mitophagy

The UPRmt and mitophagy play critical roles in repairing unfolded proteins and eliminating damaged mitochondria, respectively. However, the endogenous UPRmt may be insufficient in repairing mitochondrial damage, resulting in inevitable mitophagy activation under stress conditions, regardless of the UPRmt status [35]. An antagonistic relationship between the UPRmt and mitophagy is suggested by the inhibitory effects of mitophagy-related proteins on UPRmt-associated proteins [36]. Conversely, recent studies have examined specific stressors that stimulate the UPRmt and mitophagy simultaneously, such as muscle denervation, which induces the UPRmt and mitophagy-related protein activity [37], and high-fat diet-induced acute insulin resistance, which synchronously increases the UPRmt and mitophagy-related protein expression in mouse skeletal muscle [38]. The overexpression of mutant ornithine transcarbamylase in HeLa cells activates the UPRmt and mitophagy without affecting the membrane potential [39] and disrupted mitochondrial protein import and membrane depolarization also trigger both the UPRmt and mitophagy pathways [40,41].

Furthermore, both the UPRmt and mitophagy systems can compensate for each other when one is inadequate. For instance, the knockdown of UPRmt proteins, including HSP60, mtHSP70, and LONP1, induces the activation of mitophagy in various tissues [42,43,44,45]. Similarly, the inhibition of ATFS-1 in worms carrying mitochondrial DNA (mtDNA) mutations results in increased expression of Pink-1, a homolog of PINK1 [46]. Therefore, an accumulation of unfolded proteins and loss of mitochondrial proteostasis may increase the number of dysfunctional mitochondria that become targets for ubiquitination and mitophagy, which demonstrates that mitophagy compensates for an insufficient UPRmt process. Conversely, defective mitophagy triggers the UPRmt. In worm models of Parkinson’s disease, Pink-1 mutations lead to ATFS-1-dependent UPRmt activation [47]. In such circumstances, inefficient mitophagy can induce the accumulation of dysfunctional mitochondria, which triggers defective proteostasis and thus promotes activation of the UPRmt. At present, there is a lack of research evidence to prove whether UPRmt and mitophagy provide similar levels of compensation to each other. Therefore, this subject is worthy of further study. Overall, this coordination mechanism enables induction of the UPRmt and mitophagy simultaneously with the aim of coping with stress and preserving intraorganellar proteostasis while compensating for each other’s inefficiencies in maintaining mitochondrial homeostasis.

### 3.2. Molecular Signaling Regulates the Coordination of the UPRmt and Mitophagy

#### 3.2.1. FUNDC1

The FUN14 domain-containing 1 (FUNDC1) receptor plays a crucial role in hypoxia-induced mitophagy at the OMM [48]. Upon dephosphorylation at Ser13, FUNDC1 becomes activated and triggers hypoxia-induced mitophagy [49]. Defective mitophagy due to the loss of FUNDC1 results in the accumulation of swollen and abnormal mitochondria increased cytosolic mtDNA, and caspase-1 activation [50]. In a mouse model of myocardial ischemia/reperfusion, UPRmt activation, and inhibition alleviate and aggravate ischemia/reperfusion-mediated damage, respectively, even under slight activation of the UPRmt. Notably, the knockout of FUNDC1 in this model downregulates the expression of UPRmt-related mtDNA genes, such as CLpP, LONP1, and HSP10, whereas FUNDC1-transgenic mice show upregulated expression of these genes, indicating that FUNDC1 mediates the coordination of the UPRmt and mitophagy and may act upstream of the UPRmt pathway [51]. Moreover, ATF5 knockout-induced UPRmt depression reverses the cardioprotection induced by UPRmt activation, which leads to myocardial infarction area expansion and myocardial dysfunction [52]. This finding suggests that FUNDC1 may mediate UPRmt activation by ATF5. Additionally, Li et al. demonstrated that under proteasome inhibition stress, phosphorylated FUNDC1 interacts with HSC70, a member of the heat shock protein family, to promote the mitochondrial translocation of cytosolic unfolded proteins [53]. FUNDC1 is believed to play a role in stabilizing LONP1, as evidenced by observations of LONP1 misfolding in prostate adenocarcinoma cells upon loss of FUNDC1; recombinant LONP1 also fails to restore the reduced catalytic activity of LONP1, which suggests that FUNDC1 may promote LONP1 activity and thus facilitate the subsequent degradation of unfolded proteins [54]. Additionally, under proteasome inhibition, the knockdown of LONP1 increases the formation of mitochondrion-associated protein aggregates (MAPAs) containing FUNDC1, which indicates that the mitochondrial accumulation of LONP1 may trigger the formation of MAPAs and that FUNDC1 may participate in this process. Furthermore, the knockdown of the mitochondrial fission-related protein FIS1 also increases the number of MAPAs, suggesting that the formed MAPAs may become segregated from mitochondria via FIS1. Moreover, the knockdown of FUNDC1 inhibits the degradation of MAPAs that had formed after proteasome inhibition, but these effects are reversed by the reintroduction of FUNDC1, which indicates that FUNDC1 could promote the degradation of MAPAs. Thus, FUNDC1 plays a role not only in regulating the coordination of the UPRmt and mitophagy but also in maintaining cytoplasmic protein homeostasis under stress conditions.

#### 3.2.2. CHCHD2

CHCHD2, which is also known as mitochondrial–nuclear retrograde regulator 1, has been suggested as a stress-responsive protein in the IMS that enhances mitochondrial respiration by binding to COX. CHCHD2 is responsive to various cellular stresses, leading to its relocation from mitochondria to the nucleus, where it binds to the promoter of hundreds of genes, similar to ATF5, to boost their transcription [55]. In DW7 cells with mtDNA mutations linked to mitochondrial encephalomyopathy, CHCHD2 is decreased, whereas the enforced expression of CHCHD2 results in increases in the mitochondrial volume, mtDNA content, mitochondrial respiration, cellular levels of ATP, and ROS scavenging. These changes coincide with the simultaneous upregulation of UPRmt-related HSP60, YME1L1, and LONP1 expression and mitophagy-related Parkin expression as well as the increase in the LC3-II/I ratio induced by CHCHD2 [56]. Taken together, CHCHD2 holds promise as a mediator of the synergy between the UPRmt and mitophagy to promote mitochondrial function.

#### 3.2.3. SIRT1

Sirtuins, a highly evolutionarily conserved family of deacetylases, primarily depend on NAD^+^ levels. SIRT1 is located in both the nucleus and cytoplasm. Mouchiroud et al. showed that NAD^+^ precursor supplementation or SIRT1 overexpression in primary mouse hepatocytes induces classical UPRmt activation in a SIRT1-dependent manner, indicating the regulatory role of SIRT1 in the UPRmt [57]. SIRT1 is known to deacetylate and activate PGC-1α, and the PGC-1α-ATF5 axis has been shown to play a role in the cardioprotective effects of the UPRmt [58]. Thus, it is plausible that SIRT1 modulates UPRmt activity by regulating the PGC-1α-ATF5 pathway. Moreover, SIRT1 inactivation prevents nicotinamide-induced mitophagy in human fibroblast cells [59]. Additionally, Kume et al. demonstrated that SIRT1-induced deacetylation of Forkhead box protein transcription factor 3a (FOXO3a) promotes BNIP3 expression in hypoxic kidney environments in aging mice, indicating that SIRT1 regulates mitophagy via FOXO3a activation [60]. Thus, SIRT1 is a stress-sensitive acetylation enzyme that regulates the UPRmt and mitophagy.

#### 3.2.4. SIRT3

In contrast to SIRT1, SIRT3 is a highly prominent mitochondrial deacetylase that exerts its effects on all aspects of mitochondrial functions, such as mitochondrial fission, transcription and translation, DNA and RNA processing, lipid and amino acid metabolism, the tricarboxylic acid (TCA) cycle and OXPHOS, and confers protection against oxidative stress [61]. Furthermore, SIRT3 is a multifunctional protein that plays a role in stabilizing heterochromatin and antagonizing cellular senescence in the nucleus of human mesenchymal stem cells [62]. An increasing number of studies have demonstrated that SIRT3 is a critical regulator of the UPRmt and mitophagy. For example, primary hepatocytes lacking SIRT3 exhibit downregulated UPRmt indicator expression [63]. Lu et al. showed that SIRT3 deficiency increases the acetylation of HSP10 at Lys56, which reduces the dynamic interaction between HSP10 and HSP60 and results in increased misfolding of mitochondrial proteins, suggesting that SIRT3 regulates the UPRmt mainly in the form of HSP10/HSP60 [64]. Additionally, Yu et al. demonstrated that SIRT3 deficiency exacerbates the streptozotocin-induced impairment of mitophagy and dysfunction in cardiomyocytes due to suppressed SIRT3-FOXO3a-Parkin signaling [65]. SIRT3 is a key player in the antioxidant pathway of the UPRmt and may also act as a synchronous activator of the UPRmt and mitophagy through deacetylation and thereby providing essential protection to mitochondria.

#### 3.2.5. eIF2α

Phosphorylation of the alpha subunit of eukaryotic initiation factor 2 (eIF2α) at Ser51, which is a critical component of the ISR, is responsible for regulating translation initiation in response to environmental stress and is vital for organismal resilience. This activation of P-eIF2α reduces global mRNA translation while simultaneously enhancing the expression of stress response genes such as amino acid metabolism and apoptosis pathways. This phenomenon creates an opportunity for the cell to repair itself and facilitate the restoration of protein homeostasis. The four main kinases that activate P-eIF2α are heme-regulated inhibitor (HRI), general control nonderepressible 2 (GCN2), protein kinase R (PKR), and PKR-like endoplasmic reticulum kinase (PERK). HRI and GCN2 are activated by amino acid deficiency, whereas PKR is activated by a viral infection, and PERK is activated by endoplasmic reticulum stress [66]. Emerging evidence suggests that the activation of the PKR/eIF2α/ATF5 pathway in adipocytes is an indication of the involvement of P-eIF2α in the UPRmt activation [67,68]. Similarly, studies of Parkinson’s disease models have demonstrated P-eIF2α-dependent Parkin expression and mitophagy induction through GCN2, indicating that P-eIF2α activates mitophagy [67,69]. Collectively, the results indicate that eIF2α appears to play a critical role in the coordination machinery.

#### 3.2.6. MOTS-c

The mitochondrial ORF of the 12S type c (MOTS-c) is a mitochondria-derived peptide (MDP) or a “mitokine” that plays a critical role in metabolic regulation and has the potential for health promotion and disease prevention in biomedical research. A recent study showed that endogenous MOTS-c is secreted from exercising muscles in humans [70]. Moreover, mitochondrial ribosomal stress applied to POMC neurons induces MOTS-c in POMC neurons and nonautonomous activation of the UPRmt in adipose tissue, improving metabolism and increasing resistance to obesity in mice. Thus, MOTS-c potentially drives stress protection in neighboring cells or distant tissues through a noncell-autonomous UPRmt process. HSF1 knockdown has been found to reverse the protective effects of MOTS-c on glucose restriction and serum deprivation in myoblasts, which suggests that MOTS-c may exert its protective effects via the HSF-1-mediated UPRmt. Furthermore, MOTS-c treatment has been found to enhance mitophagy in the liver and reduce the accumulation of abnormal lipid deposits caused by mild mitochondrial stress [71], further supporting the notion that MOTS-c plays a role in regulating mitophagy. Therefore, as a systemic signaling molecule, MOTS-c has the potential to simultaneously activate the UPRmt and mitophagy.

#### 3.2.7. FGF21

The hormone fibroblast growth factor 21 (FGF21) is a mitokine that ameliorates metabolic dysfunction, reduces body weight, and confers systemic protection against various diseases [72]. Although its expression in healthy muscle is low, the release of FGF21 from muscle is induced by certain stressors, such as fasting, cold, and exercise. The knockout of skeletal muscle FUNDC1 and LONP1 results in increased FGF21 release into the blood circulation, which induces the UPRmt and metabolic remodeling in adipose tissue, thus resisting obesity and improving systemic metabolism [73,74]. Moreover, akin to the UPRmt, FGF21 has been implicated in antiaging effects, particularly in the extension of lifespan induced by dietary protein restriction [75]. Additionally, studies have shown that FGF21 deficiency leads to impaired BNIP3L-mediated mitophagy in skeletal muscle [76], potentially mediated by AMPK activation [77]. These results suggest that FGF21 may regulate the coordination of the UPRmt and mitophagy to protect against stress.

**Figure 3:**
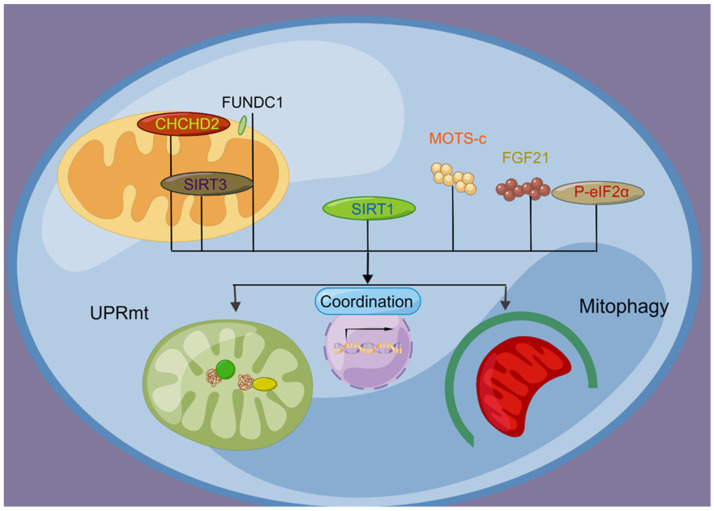
Molecular signaling coordinates the UPRmt and mitophagy. Upstream molecular signaling molecules, including FUNDC1, CHCHD2, SIRT1, SIRT3, eIF2α, MOTS-c, and FGF21, regulate the coordination of the UPRmt and mitophagy to repair and remove damaged mitochondria.

In summary, the UPRmt and mitophagy are regulated through shared upstream molecular signaling pathways, which underlie their coordinated action in ensuring mitochondrial homeostasis (Figure 3).

## 4. MQC Dysfunction in Aging Skeletal Muscle

Aging muscle is characterized by defective organelles, such as increased levels of mutant mtDNA (ΔmtDNA), higher susceptibility to mitochondrial apoptosis, and increased oxidative stress [78,79]. These changes may be attributed to disrupted MQC. In addition to impaired mitochondrial biogenesis, studies have reported increased mitochondrial fragmentation, whereas others have observed mitochondrial hyperfusion in aging muscle [80,81]. Mitochondrial fragmentation resulting from decreased fusion and/or increased fission can compromise the mtDNA integrity and mitochondrial structural and functional complementation, leading to increased ROS production. Conversely, the formation of enlarged mitochondria due to decreased fission and/or increased fusion can impair mitophagy and biogenesis, resulting in the accumulation of damaged mitochondria in aging cells.

### 4.1. UPRmt Abnormalities in Aging Skeletal Muscle

The impact of aging on the UPRmt has become increasingly evident, and research shows that the UPRmt is suppressed during the aging process. In skeletal muscle from aging mice, the levels of UPRmt proteins are reduced, and in muscle biopsy samples from sarcopenic humans, the expression of UPRmt genes is decreased, indicating an insufficient UPRmt response to repair or neutralize oxidative stress-induced damage to mitochondrial proteins [82,83]. Conversely, an elevated UPRmt can promote senescence resistance, as evidenced by the finding that the reactivation of the UPRmt inhibits muscle stem cell senescence in aging mice [84]. Studies have also shown that LONP1, a mitochondrial protease of the classical UPRmt pathway, is reduced in aging skeletal muscle [45]. Notably, muscle-specific ablation of LONP1 exacerbates denervation-induced muscle loss, and this is likely due to the overaccumulation of mitochondrial unfolded proteins activating the autophagy-lysosome degradation program. These results demonstrate the essential role of LONP1 in safeguarding mitochondrial function and preserving muscle mass and highlight the detrimental effects of aging-related oxidative stress on the UPRmt, which ultimately lead to sarcopenia.

Considerable evidence supports the notion that the UPRmt promotes the extension of the lifespan and health span across several model organisms. In worms, the epigenetic memory of the UPRmt induced by mitochondrial stress during the developmental stage is crucial for lifespan extension [85]. In this regard, L1 stage worms with stress-induced UPRmt exhibit a reduction in lifespan upon HDA-1 (histone deacetylase homolog) silencing [86]. However, in the L4 stage, mitochondrial stress robustly activates HSP-6p (HSP60 homolog in mitochondria) and thereby almost doubles the lifespan of worms [87]. Intriguingly, the deletion of a specific F-ATP synthase subunit (complex V in mitochondria) increases lifespan when initiated during larval development but curtails it when initiated during adulthood [88]. Moreover, excessive stress during worm development results in a shorter lifespan [89]. These findings underscore the criticality of inducing mitochondrial stress during L4 development within a narrow range of stress levels for longevity extension via the UPRmt. Additionally, the lifespan extension induced by the UPRmt can entail several detrimental consequences, including growth retardation, reduced body size, and impaired fertility [87,90]. In worms characterized by 60% ΔmtDNA, UPRmt inhibition reduces the transmission of deleterious ΔmtDNA, whereas UPRmt activation increases the total mtDNA copy number, including both wild-type and deleterious ΔmtDNA [91]. This outcome is likely due to the sustained UPRmt-induced mitochondrial biogenesis that leads to the accumulation of ΔmtDNA. Additionally, prolonged UPRmt activation can induce harmful metabolic remodeling, which can benefit cancer cell survival [92,93]. Cytotoxicity related to metabolic remodeling may be mediated by ATF5 because the overexpression of ATF5 has been observed to increase protein catabolism-related metabolites and thereby reduce muscle mass in mice [94]. Thus, short-term activation of the UPRmt is protective, but a sustained UPRmt under oxidative stress promotes sarcopenia. The downregulation of the UPRmt during aging may therefore be an evolutionarily selected mechanism.

### 4.2. Mitophagy Aberrations in Aging Skeletal Muscle

Aging tissues are known to accumulate severely damaged organelles, particularly mitochondria. Mitophagy, the process by which damaged mitochondria are cleared, is essential for the maintenance of MQC. Although decreased mitophagy has been directly linked to age-related declines in cardiac and neural organs, the precise impact of aging on mitophagy in skeletal muscle remains unclear. Conflicting evidence has been reported: some studies indicate an increase in mitophagy, and others suggest a decrease. Possible explanations for these inconsistencies include differences in the measurement methods and the notion that an increase in mitophagy may serve compensatory functions but does not necessarily confer oxidative stress resistance because the number of damaged mitochondria continues to increase with age [6]. Evidence supporting the decrease hypothesis includes findings showing that increased or unchanged mitophagy pathways are accompanied by a reduced ratio of Parkin to voltage-dependent anion channel (VDAC) levels in aging muscle, which indicates a substantial decrease in mitophagy [95]. Moreover, studies suggest reduced expression of key mitophagy regulators such as PINK1/Parkin, BNIP3, Nix, and LC3 and increased lysosomal accumulation of lipofuscin in aging skeletal muscle [14,96,97]. These mitophagy defects are accompanied by decreased mitochondrial size and muscle mass loss. In recent years, Parkin has emerged as a critical player in the defense against mitochondrial dysfunction and muscle atrophy. Evidence shows that Parkin deficiency disrupts mitophagy and contributes to chronic obstructive pulmonary disease, and these effects are accompanied by increased mitochondrial ROS levels and muscle atrophy [98]. Furthermore, Parkin-deficient muscle shows impaired mitochondrial respiration, mitochondrial fragmentation, and sensitization of the mitochondrial permeability transition pore [99]. Conversely, the overexpression of Parkin mitigates sepsis-induced muscle atrophy by increasing mitochondrial quality and content as well as the muscle mass and strength in aging muscles [100]. These findings underscore the pivotal role of Parkin in maintaining mitochondrial function and preventing muscle atrophy during aging. Future studies should explore the possibility of targeting Parkin as an intervention strategy to prevent sarcopenia.

### 4.3. The Coordination Mechanism in Aging Skeletal Muscles Is Impaired

During cellular senescence, dysfunction of the MQC system is mainly caused by oxidative stress and redox imbalance, which leads to a breakdown of the coordination mechanism and contributes to mitochondrial dysfunction in aging organs. Although the effect of aging on FUNDC1 expression in skeletal muscle is unknown, its downregulation during aging has been reported in other organs, such as the myocardium [98]. Additionally, a reduction in CHCHD2 levels has been observed in mitochondrial encephalomyopathy-related mutant cells, leading to impairments in the UPRmt and mitophagy [56]. In aging skeletal muscle, the levels of SIRT1, a key regulator of mitochondrial function, are decreased and associated with reduced AMPK activity [101]. Furthermore, SIRT1 activity is compromised due to a systemic decline in NAD^+^ levels in the cytosol and nucleus of aging skeletal muscle [102]. In cardiomyocytes of aged rats, the downregulation of the SIRT1-PINK1-Parkin pathway leads to a reduction in mitophagy [103]. Similarly, our unpublished research showed that the skeletal muscle of old mice exhibits downregulated SIRT3 expression and decreased levels of HSP60. Furthermore, SIRT3 deficiency has been found to impair Parkin-mediated mitophagy in cardiomyocytes by blocking the mitochondrial translocation of Parkin [104]. Interestingly, the level of P-eIF2α is increased in aging muscle, suggesting a complex context that requires further elucidation [105]. The levels of the mitokine MOTS-c are decreased in the skeletal muscle and circulatory system of aging humans and mice, whereas FGF21 levels are increased in aging plasma [106,107,108]. The differences among mitokines require further investigation. Overall, aging leads to decreased levels of FUNDC1, CHCHD2, and MOTS-c and inactivated SIRT1 and SIRT3, which may impair the coordination mechanism function.

In summary, sarcopenia may be caused by a dysfunctional coordination mechanism that disrupts both the UPRmt and mitophagy, which ultimately compromises organelle homeostasis.

## 5. Exercise Maintains Mitochondrial Health in Aging Skeletal Muscle by Enhancing the UPRmt and Mitophagy

Physical activity and exercise have been proposed as effective interventions for improving the quality of aging muscles or delaying the onset of sarcopenia. In particular, aerobic exercise has been shown to partially alleviate sarcopenia-related issues caused by mitochondrial dysfunction, including apoptosis, mitochondrial oxidative stress, myostatin, and inflammatory cytokines [109]. Moreover, endurance training has been implicated in maintaining MQC and may therefore contribute to the prevention and management of sarcopenia during aging [5].

### 5.1. Exercise and the UPRmt

To date, numerous studies have demonstrated that exercise effectively activates the UPRmt pathway in skeletal muscle. For instance, the Hood research group utilized chronic contractile activity to simulate exercise and observed increased levels of UPRmt-related mRNA and proteins in rat skeletal muscle, and similar results have been observed in murine myocytes [110,111]. Additionally, studies have found that ATF5 plays a critical role in exercise-induced UPRmt and mitochondrial homeostasis because ATF5-knockout muscle displays an enhanced exercise-induced stress kinase signaling response but a blunted UPRmt and mitophagic gene expression response [112]. More recently, our group found that just 12 days of exercise is sufficient for increasing the expression of c-Jun, HSP60, and CLpP in mouse skeletal muscle, which strongly suggests the activation of the classical UPRmt pathway [113]. These results collectively highlight the effectiveness of exercise in promoting the activation of the UPRmt pathway in skeletal muscle.

In addition, exercise has been shown to counteract the decline in the UPRmt observed with aging. In mouse gastrocnemius muscle, the mRNA levels of UPRmt-related genes, such as Yme1L1 and CLpP, decrease with aging; however, 4 weeks of aerobic exercise increase the expression of these factors, in line with an increase in mitochondrial content [82]. Similarly, 24-month-old mice exhibit an improved response to 4 weeks of high-intensity interval training, which leads to increased mRNA levels of Yme1L1 and LONP1 and heightened protein levels of LONP1 in skeletal muscle. These effects consist of enhanced mitochondrial quality, as indicated by increased PGC-1α and citrate synthase levels, and improved physical performance including increases in grip strength, maximum running speed, and running distance [13]. These findings provide evidence showing that exercise can trigger classical UPRmt pathway activation under normal physiological conditions and reverse age-related UPRmt disruption.

### 5.2. Exercise and Mitophagy

Acute exercise has been shown to induce mitophagy in skeletal muscle during the recovery period through AMPK-mediated phosphorylation of ULK1 at Ser555 [114]. This process appears to occur independently of PINK1 stabilization on mitochondria, suggesting that PINK1 is not involved in exercise-induced mitophagy in skeletal muscle [115]. Notably, a specific isoform of AMPK, mitoAMPK, located on the OMM, has been found to contribute to acute exercise-induced mitophagy [116]. Endurance exercise has been shown to increase the mitochondrial localization of Parkin, the expression of BNIP3 and Nix, the conversion of LC3-I to LC3-II, and the flux of mitophagy in skeletal muscle [117,118,119,120]. Interestingly, six weeks of voluntary running reduces basal mitophagy in mouse muscles and weakens their mitophagy response to acute exercise, suggesting that the increase in organelle quality induced by chronic exercise leads to exercise adaptation that reduces the need for degrading damaged mitochondria [121]. Furthermore, the exercise-induced mitophagy response is abolished in Parkin-knockout skeletal muscle, which establishes Parkin as a crucial and direct regulator of exercise-induced mitophagy.

Mitophagy has emerged as a critical mechanism underlying exercise adaptation in chronic diseases and aging. Lifelong exercise training can mitigate age-related decreases in the LC3II and BNIP3 levels in human muscle, which are mediated through the PGC-1α-p53 axis [122]. The overexpression of Parkin has been shown to enhance mitochondrial clearance and protect aging skeletal muscle from metabolic decline and atrophy [14]. However, the increases in Parkin expression and mitophagy flux in the contracting muscle of 18-month-old mice have been found to be diminished, indicating that the exercise response of mitophagy declines with age [95]. The accumulation of lipofuscin in lysosomes has been observed in aging skeletal muscle and is considered a sign of decreased lysosomal activity and a rate-limiting step in mitophagy [123]. Thus, an impaired lysosomal function may contribute to the decline in the exercise response of mitophagy at advanced ages. These results suggest that long-term exercise can reverse mitophagy defects in aging, despite some cases of low response in advanced age.

### 5.3. Exercise Promotes the Coordination Mechanism of the UPRmt and Mitophagy

Although the role of FUNDC1 in exercise has not been fully explored, studies have shown that the electrical pulse stimulation of C2C12 cells increases the FUNDC1 levels, elevates the LC3-II/I ratio and reduces the p62 levels, whereas the inhibition of FUNDC1 upstream factors decreases its levels, resulting in reduced colocalization of LC3, FUNDC1, and COXIV, suggesting that FUNDC1 mediates exercise-induced mitophagy [124]. In mouse gastrocnemius muscle, 4 weeks of aerobic exercise increases SIRT1 expression, and this effect is accompanied by reduced global protein acetylation and increased expression of HSP60, LONP1, and Yme1L1 [82]. Furthermore, exercise training increases SIRT1 and SIRT3 expression in aging skeletal muscle [102]. Swimming for eight weeks increases SIRT3 and PINK1/Parkin expression and decreases the p62 levels in 18-month-old mice following stable myocardial infarction [115]. These findings support the notion that sirtuins may partially restore the coordination mechanism in aging mediated by exercise. In mouse gastrocnemius muscle, low-intensity running exercise for 90 min increases eIF2α phosphorylation at Ser51 and ATF4 gene expression [125]. Additionally, acute high-intensity aerobic exercise increases the levels of MOTS-c in skeletal muscle and plasma of healthy young males; thus, the exercise-mediated MOTS-c-HSF-1 pathway may be involved in UPRmt activation in skeletal muscle [70]. Acute exercise also increases the FGF-21 levels, which improves lipid metabolism and prevents sarcopenia, although this increase lasts only for 3 h [126,127]. Consequently, regular exercise can fine-tune and rescue coordinated molecular signaling during aging, leading to restoration of both the UPRmt and mitophagy and reversal of deteriorated organelle homeostasis in old age.

In summary, moderate-intensity muscle contraction cumulatively enhances both the UPRmt and mitophagy and consequently restores organelle homeostasis during old age through the pivotal molecular signaling pathways that regulate the coordination mechanism.

## 6. Exercise Induces ROS to Regulate the Coordination Mechanism of the UPRmt and Mitophagy

Mitochondria are considered the primary sources of ROS production during exercise, although some researchers suggest that nonmitochondrial sources, such as NADPH oxidases and xanthine oxidases, may also contribute to this effect [128]. Antioxidant defense systems tightly regulate the availability of stable ROS, such as H_2_O_2_ and NO, which often serve as redox-sensitive signals for regulating protein metabolism and mitochondrial function. Recent evidence suggests that moderate exercise enhances the redox balance by upregulating compensatory antioxidant capability, which is the main mediator of the health effects of exercise. This finding contrasts with antioxidant intervention, which inhibits oxidative stress and induces a low-level redox balance, reducing or abolishing exercise-associated stress and preventing the health-promoting effects of exercise. Our group recently demonstrated that inhibiting mitochondrial ROS generation in mice during exercise using MitoTEMPO, a novel cell-penetrating antioxidant targeting mitochondria, reduces the increases in the c-Jun, HSP60, and CLpP levels in the classical pathway of the UPRmt in skeletal muscle, indicating that exercise-derived ROS stimulate the UPRmt [113]. Additionally, an acute burst of ROS from mitochondria induced by the mitochondrial-targeted photosensitizer KillerRed results in the activation of Parkin-dependent mitophagy [129]. ROS are produced transiently and moderately in response to exercise, mediating a high level of antioxidants and likely coordinating the UPRmt and mitophagy.

**Figure 4:**
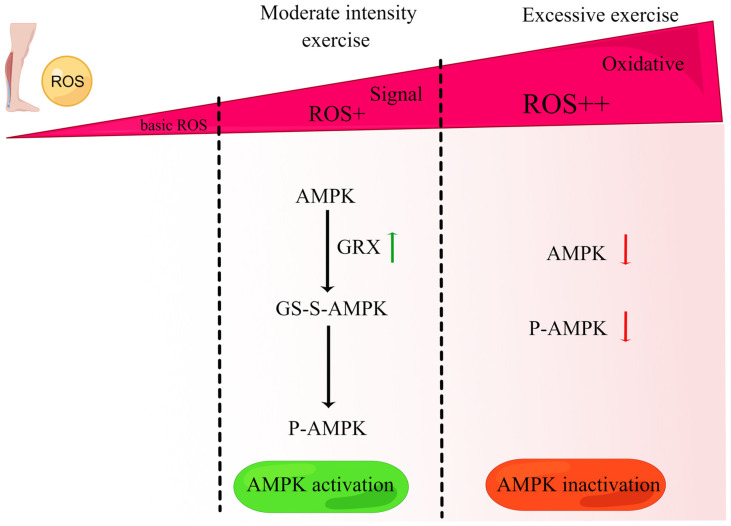
ROS bidirectionally regulates P-AMPK in exercise. In the presence of a moderate concentration of the signal H_2_O_2_ (approximately 10–100 µM), AMPK can be activated by glutaredoxin (GRX)-mediated S-glutathionylation (GS-S-) and phosphorylation (P-), leading to AMPK activation, and this is consistent with the upregulation of glutaredoxin and phosphorylated activation of AMPK caused by moderate-intensity exercise. In contrast, a relatively high concentration of H_2_O_2_ (approximately 200 µM) that generates oxidative stress results in decreases in the S-glutathionylation, phosphorylation, and protein content of AMPK, consistent with the result that excessive exercise reduces AMPK activity. Green arrows indicate proteins that are downregulated, red arrows indicate proteins that are upregulated.

The redox-sensitive kinase AMPK, activated by decreased intracellular ATP levels or increased ROS production, is a hallmark of intensive exercise in animal and human studies [130]. However, AMPK activity declines with aging [131]. Some studies suggest that exercise activates AMPK through ROS-mediated mechanisms. For instance, in L02 cells, Dong et al. demonstrated that under low H_2_O_2_ conditions (approximately 10–100 µM), AMPK is activated by glutaredoxin-mediated S-glutathionylation and phosphorylation [131]. These findings are consistent with the upregulation of glutaredoxin, phosphorylation-related activation of AMPK, and promotion of mitochondrial TCA observed in the liver of diabetic rats following moderate-intensity exercise [132]. Conversely, a relatively high concentration of H_2_O_2_ (approximately 200 µM) results in reductions in the S-glutathionylation, phosphorylation, and protein content of AMPK. This observation is consistent with evidence indicating that excessive exercise-induced oxidative stress decreases AMPK expression and disrupts the mitochondrial structure. These divergent effects of ROS on AMPK activity highlight a bidirectional regulation of AMPK by ROS (Figure 4).

Acute exercise has been demonstrated to increase AMPK phosphorylation at Thr172 and ULK1 phosphorylation at Ser555 in skeletal muscle [133]. Our previous study using mice expressing dominant negative and constitutively active AMPK in skeletal muscle confirmed that ULK1 activation and subsequent mitophagy induction in response to acute exercise relies on AMPK activation in skeletal muscle [114]. Moreover, we found that ULK1 is crucial for targeting the lysosome to damaged/challenged mitochondria during mitophagy because deletion of the ULK1 gene in skeletal muscle inhibits mitophagy in response to exercise without affecting lysosome formation. In addition, recent research has shown that the AMPK-ULK1 cascade also regulates the FUNDC1 levels during exercise: electrical pulse-stimulated C2C12 myotubes exhibit increased levels of AMPK, ULK1, and FUNDC1, and the AMPK inhibitor Compound C suppresses this pathway, leading to a decrease in the FUNDC1 levels [124]. Furthermore, Bax inhibitor-1, an antiapoptotic protein that reduces the accumulation of ROS, has been shown to activate the UPRmt and FUNDC1-mediated mitophagy in cardiorenal syndrome type 3, indicating that moderate ROS levels may be involved in the coordination mechanism, and their effects are likely mediated via FUNDC1 [134]. In addition to FUNDC1, the redox-sensitive kinase AMPK has been suggested to enhance SIRT1 activity by increasing the cellular NAD^+^ levels. Exercise-induced alteration of the NAD^+^/NADH ratio has been shown to increase SIRT1 activity in various organs [135]. Furthermore, endurance training has been found to upregulate P-AMPK and SIRT1 expression in aging skeletal muscle [101]. Based on these observations, it can be inferred that exercise activates SIRT1 through AMPK. In contrast, SIRT1 phosphorylates the Thr172 residue of the AMPK α subunit by deacetylating LKB1 in skeletal muscle [136], indicating that AMPK-SIRT1 crosstalk plays a vital role during exercise. Nevertheless, oxidative stress can reduce the SIRT1 mRNA levels by inducing microRNA expression. Baker et al. demonstrated that exogenous H_2_O_2_ (100 and 150 µM) activates miR-34a through PI3Kα in human bronchial epithelial cells, and miR-34a binds to the 3’UTR of SIRT1 mRNA, inhibiting SIRT1 expression [137]. The expression of miR-34a is elevated in aging skeletal muscle, potentially due to dysregulated SIRT1 [138]. Consequently, the AMPK-SIRT1 feedback loop is positively controlled by moderate exercise-derived ROS but negatively regulated by excessive ROS under oxidative stress conditions or during aging. SIRT3 represents a crucial downstream molecule of AMPK [139]. Phloretin, a dihydrochalcone, enhances SIRT3 expression via the phosphorylation of AMPKα2 at Thr172 in human umbilical vein endothelial cells and mouse aortas [140]. Conversely, the inhibition of AMPKα2 attenuates the increase in SIRT3 expression induced by exercise in mouse skeletal muscle [141]. The activation of SIRT3 by AMPK also depends on NAD^+^ levels. In vivo studies have demonstrated that a decline in NAD^+^ levels can impair SIRT3 activity, leading to mitochondrial dysfunction and redox disruption due to the acetylation of key enzymes and transcription factors [142,143]. Moreover, under proteotoxic stress conditions, the expression of SIRT3 is abolished by treatment with the antioxidant NAC, implying that ROS upregulate SIRT3 expression under stress conditions [144]. Therefore, exercise-induced moderate ROS production may activate SIRT3 through AMPK activation. A previous study demonstrated that AMPK-activated SIRT3 deacetylates YME1L1 and thereby suppresses its protease activity on OPA1 cleavage, leading to an increase in the OPA1 levels, which suggests that the AMPK-SIRT3 pathway may act as an inhibitor of YME1L1 protease activity [145]. YME1L1 is crucial for the specific degradation of CHCHD2 in mitochondria under mild stress conditions [56]. These findings indicate that ROS may indirectly regulate CHCHD2 via the AMPK/SIRT3/YME1L1 pathway. AMPK also plays a key role in regulating the activity of P-eIF2α. Treatment with AMPK agonists or inhibitors upregulates or downregulates the AMPK/PERK/P-eIF2α signaling pathway, respectively [146]. Acute exposure to H_2_O_2_ upregulates the P-eIF2α/eIF2α and CHOP levels in C2C12 myoblasts [147]. Under moderate hypoxia, ROS promote P-eIF2α activity, which promotes energy and redox homeostasis and enhances cell survival [148]. Thus, ROS play a crucial role in the AMPK-P-eIF2α signaling pathway.

Recent research has uncovered the involvement of mitokines, specifically the exercise-induced translocation of N1 hypothalamic neuronal MOTS-c to the nucleus for gene transcription. Interestingly, the regulation of MOTS-c appears to be dependent on ROS produced at moderate levels, as evidenced by the reversal of MOTS-c translocation upon treatment with the antioxidant N-acetylcysteine [149,150]. Fu et al. demonstrated that the skeletal muscle-specific ablation of FUNDC1 upregulates FGF21 expression in muscle, which in turn acts as an interorgan communication signal, leading to thermogenesis remodeling in adipose tissue [74]. The study further revealed that the potent antioxidant Trolox completely abolishes FUNDC1 knockout-induced FGF21 expression and thus demonstrates the crucial role of the ROS-induced retrograde response in regulating FGF21. These results provide a compelling link between ROS and mitokines.

Exercise-derived ROS act as molecular signals of exercise adaptation that mediate the coordination of the UPRmt and mitophagy through the AMPK-mediated redox-sensitive pathways discussed above.

## 7. Conclusions

**Figure 5:**
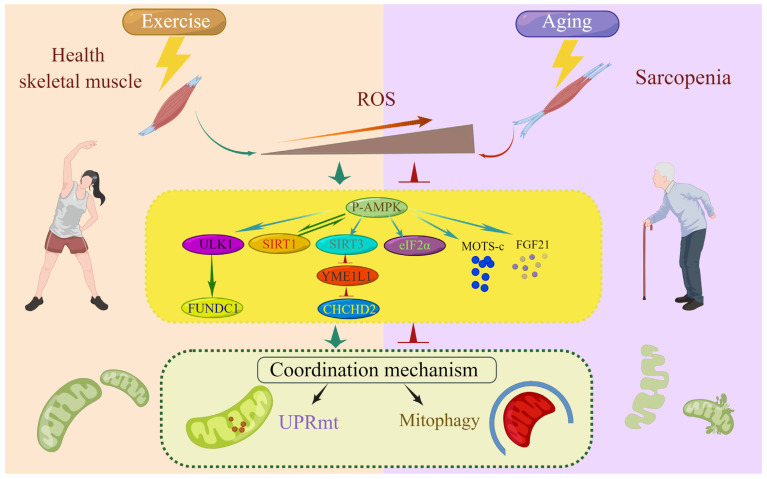
Proposed model of ROS regulation of the coordination mechanism of the UPRmt and mitophagy with exercise and during aging. As redox signals of exercise adaptation, ROS upregulate the coordination of the UPRmt and mitophagy through the AMPK/ULK1/FUNDC1, AMPK/SIRT1, and AMPK/SIRT3/YME1L1/CHCHD2 pathways, P-eIF2α, MOTS-c, and FGF21, underlying the health benefits of exercise. During aging, excessive ROS induce prolonged oxidative stress, downregulating these signaling pathways and leading to a coordinated decrease in the UPRmt and mitophagy, which disrupts mitochondrial homeostasis and causes sarcopenia.

Despite considerable progress toward understanding the classical regulators of the UPRmt and mitophagy, the underlying crosstalk mechanisms remain incompletely understood. The coordination mechanism mediates the activation of both processes under certain conditions, allowing them to compensate for each other when either process is insufficient and thus enhancing mitochondrial homeostasis and vitality. Multiple regulatory signaling pathways mediate this coordinated response, allowing mitochondria to adapt to physiological stress and maintain muscle function. However, this mechanism is attenuated in aging skeletal muscle, leading to abnormal mitochondrial homeostasis and sarcopenia development. Exercise restores the coordination mechanism and subsequent UPRmt and mitophagy to mitigate mitochondrial dysregulation in aging and sarcopenia. The concept of “ROS hormesis” describes how mild exercise-induced redox-sensitive signaling can result in beneficial adaptations that protect against more severe stresses, such as aging. The reductions in physical activity and cellular function during aging can trigger oxidative stress and sarcopenia. However, moderate and transient ROS production during exercise appears to be beneficial. Aging-induced ROS inhibit AMPK and its downstream signaling pathways and thereby suppress the coordination mechanism. Exercise-derived ROS activate AMPK, stimulating coordination, stabilizing mitochondrial homeostasis in aging, and preventing or reversing sarcopenia. The bidirectional regulation of the coordination mechanism by ROS and AMPK highlights the regulatory networks coordinating MQC (Figure 5). Identifying the molecular targets involved in this coordination mechanism may provide valuable insights into the development of therapeutic strategies for the treatment of sarcopenia and for promoting muscle health throughout the human lifespan.

## Data Availability

Not applicable.
